# Author Correction: Three-phase electric power driven electroluminescent devices

**DOI:** 10.1038/s41467-021-20976-0

**Published:** 2021-01-18

**Authors:** Junpeng Ji, Igor F. Perepichka, Junwu Bai, Dan Hu, Xiuru Xu, Ming Liu, Tao Wang, Changbin Zhao, Hong Meng, Wei Huang

**Affiliations:** 1 School of Advanced Materials, Peking University Shenzhen Graduate School, 2199 Lishui Road, Shenzhen, 518055 China; 2 Institute of Flexible Electronics, Northwestern Polytechnical University, 127 West Youyi Road, Xi’an, 710072 China; 3 College of Mechatronics and Control Engineering, Shenzhen University, 3688 Nanhai Street, Shenzhen, 518000 China

**Keywords:** Optical sensors, Imaging and sensing

Correction to: *Nature Communications* 10.1038/s41467-020-20265-2, published online 4 January 2021.

The original version of this Article contained an error in the title, which was previously incorrectly given as ‘Three-phase electric power driven electoluminescent devices’. The correct version states ‘electroluminescent’ in place of ‘electoluminescent’.

This has been corrected in both the PDF and HTML versions of the Article.

