# Supplementary material for: Three-phase electric power driven electoluminescent devices
Source: Nat Commun. 2021 Jan 4;12:54. doi: 10.1038/s41467-020-20265-2 (PMC7782587; doi:10.1038/s41467-020-20265-2)
Supplement: Supplementary file 2 — Supplementary Information [file 41467_2020_20265_MOESM2_ESM.pdf]

Supplementary Information

Three-phase electric power driven electroluminescent devices

Junpeng Ji[1], Igor F. Perepichka[2], Junwu Bai[1], Dan Hu[1], Xiuru Xu[3], Ming Liu[1], Tao Wang[1], Changbin Zhao[1], Hong Meng[1]*, Wei Huang[2]*

[1] School of Advanced Materials, Peking University Shenzhen Graduate School, Shenzhen 518055, China.

[2] Institute of Flexible Electronics, Northwestern Polytechnical University, 127 West Youyi Road, Xi'an 710072, China.

[3] College of Mechatronics and Control Engineering, Shenzhen University, 3688 Nanhai Street, Shenzhen 518000, China.

*Corresponding author. E-mail: menghong@pku.edu.cn (H.M.), iamwhuang@nwpu.edu.cn (W.H.)

Table of Contents pages

1. Supplementary Note 1:
   The reasons why the three-phase power supply system is in use globally…… S2
2. Supplementary Note 2:
   Distribution of electric field intensity
   between the phosphor layer and the dielectric layer …………………… S2
3. Supplementary Note 3:
   The relationship between the voltage and the current in a composite circuit … S3
4. Supplementary Note 4:
   Performance of TP-OLEDs ……………………………………………………… S4
5. Supplementary Figures 1 – 21 ………………………………………………………… S5–S22

1. Supplementary Note 1: The reasons why the three-phase power supply system is in use globally.

In terms of power generation, a three-phase generator of the same size produces higher power than a single-phase generator. In the case of a three-phase balanced load, the generator torque is constant, which is beneficial for operation of the generator. In terms of power consumption, a three-phase electric power easy generates a rotating magnetic field making a three-phase motor to rotate smoothly. Finally, in terms of transmission, the three-phase system uses fewer wires compared to the single-phase system and the ratio of a conductor material to capacity is halved by using TP system.

Importantly, it can be verified in theory that when the electric transmitting system for loads of equal capacity is changed from single-phase system to three-phase system, the line loss can be reduced by 6 times (when the three-phase load reaches equilibrium), as described below.

Assume that the three-phase load reaches equilibrium, all conductive wires are the same and the resistance of a single conductive wire is R, the current values in a single-phase power transmitting system and in a three-phase four-wire transmitting system are denoted by I_{single} and I_{three}, respectively. When the total power of the load in a single-phase power transmitting system is equal to that in a three-phase power transmitting system, the relationship between I_{single} and I_{three} follows Supplementary eq. 1:

$$I_{single} = 3I_{three} \quad \text{(Supplementary eq. 1)}$$

When the three phases are balanced, the current in the neutral line is zero. Therefore, the line loss (W_{single}) of two conductive wires in a single-phase power transmitting system is expressed by Supplementary eq. 2:

$$W_{single} = 2\cdot I_{single}^2\cdot R = 2\cdot(3I_{three})^2\cdot R = 18\cdot I_{three}^2\cdot R \quad \text{(Supplementary eq. 2)}$$

where R is the resistance of a single conductive wire.

At the same time, the total line loss when using a three-phase four-wire transmitting system (W_{three}) is described by Supplementary eq. 3:

$$W_{three} = 3\cdot I_{three}^2\cdot R \quad \text{(Supplementary eq. 3)}$$

Therefore, the line losses in a single-phase power transmitting system are 6 times higher of that in a three-phase transmitting system.

2. Supplementary Note 2: Distribution of electric field intensity between the phosphor layer and the dielectric layer.

The TPEL device can be simulated by a two-layer dielectric model under uniform electric field as shown in Supplementary Fig. 2 (a,b). The thickness, dielectric constant and electric field intensity of the phosphor layer are denoted as d_1, ε_1 and E_1, respectively. Similarly, the thickness, dielectric

constant and electric field intensity of the dielectric layer are d_2, ε_2 and E_2, respectively. In such two-layer dielectric model under uniform electric field, the relationship Supplementary eq. 4 is true and the total voltage U in the two-layer dielectric model can be expressed by Supplementary eq. 5:

$$\varepsilon_1 \cdot E_1 = \varepsilon_2 \cdot E_2 \quad \text{(Supplementary eq. 4)}$$

$$U = E_1 \cdot d_1 + E_2 \cdot d_2 \quad \text{(Supplementary eq. 5)}$$

Therefore, the distribution of the electric field intensity between the phosphor layer and the dielectric layer can be easily deduced as a function of ε_1, ε_2 and d_1, d_2 (Supplementary eq. 6 and Supplementary eq. 7):

$$E_1 = \frac{U}{\varepsilon_1\left(\frac{d_1}{\varepsilon_1}+\frac{d_2}{\varepsilon_2}\right)} \quad \text{(Supplementary eq. 6)}$$

$$E_2 = \frac{U}{\varepsilon_2\left(\frac{d_1}{\varepsilon_1}+\frac{d_2}{\varepsilon_2}\right)} \quad \text{(Supplementary eq. 7)}$$

In order to increase the electric field intensity in the phosphor layer, we need the dielectric layer to be thinner and/or with higher dielectric constant.

3. Supplementary Note 3: The relationship between the voltage and the current in a composite circuit.

Suppose the voltage applied to the capacitor as a function of time (t) follows the Supplementary eq. 8:

$$V(t) = A\cos(\omega t) \quad \text{(Supplementary eq. 8)}$$

Then, the current can be expressed by Supplementary eq. 9:

$$I(t) = C\frac{dV}{dt} = -C\omega A\sin(\omega t) = C\omega A\cos\left(\omega t + \frac{\pi}{2}\right) \quad \text{(Supplementary eq. 9)}$$

Thus, the current in a capacitance only circuit is always 90 ° ahead of the voltage applied. However, our measurements (Fig. 2a) showed the current to be 35 ° ahead of the voltage applied (at 53 V_{rms}, 1 kHz) instead of 90 °, indicating that there is also a resistive component in the circuit, in addition to the capacitive component. The impedance caused by the capacitive component is 90 ° out of the impedance caused by the resistive component and can be regarded as an imaginary component, forming an impedance triangle as shown in Supplementary Fig. 2c. From the impedance triangle, it is clear seen that in a composite circuit including capacitive component and resistive component, the angle between the current and voltage (α) can be expressed by Supplementary eq. 10:

$$\alpha = arctan\frac{-X_C}{R} = arctan\frac{-U_C}{U_R} \quad \text{(Supplementary eq. 10),}$$

where X_C is capacitive reactance of the circuit, U_C and U_R are the voltage values across the resistor part and capacitance part, respectively.

Negative sign in Supplementary eq. 10 indicates that the current is ahead of the voltage. So, a 35° ahead of current relatively to applied voltage indicates that in our TPEL devices, the ratio of the resistive component to the capacitive component in impedance (X_C/R) is ca. 0.7, exhibiting capacitive and resistive properties at the same time.

4. Supplementary Note 4: Performance of TP-OLEDs

Fig. 6c shows the normalized EL spectra of the TP-OLEDs with different dopants in the EML layers to provide different color emission from the devices. The emission peaks of the devices are: λ_{EL} = 456 nm (blue), 473 nm (cyan-blue), 524 nm (green) and 616 nm (red). The configuration of the devices presented in Fig. 6 are as follows:

Green: HATCN (10 nm) / HTM (30 nm) / 48.8% H1 : 48.8% H2 : 2.4% GD (50 nm) / ETM (30 nm) / Liq (2.5 nm) / Al (100 nm)

Blue: HATCN (10 nm) / HTM (30 nm) / 90.9% H3 : 9.1% BD (50 nm) / ETM (30 nm) / Liq (2.5 nm) / Al (100 nm)

Cyan-blue: HATCN (10 nm) / TCTA (40 nm) / 90.9% 26DCzPPy : 9.1% FIrpic (50 nm) / BPhen (40 nm) / Liq (2.5 nm) / Al (100 nm)

Red: HATCN (10 nm) / HTM (30 nm) / 65% H1 : 32.5% H2 : 2.5% RD (40 nm) / ETM (30 nm) / Liq (2.5 nm) / Al (100 nm)

The electroluminescent performance and efficiency of TP-OLEDs with different EMLs as functions of applied voltage are shown in. Fig. 6d-f. Both the luminance intensity and the current density increased with the applied voltage at a fixed frequency of 10 kHz, reaching maximum luminance of 390, 1193, 6277 and 2112 cd/m^2, for blue, cyan-blue, green and red TP-OLED devices, respectively (Fig. 6d). The current efficiencies of TP-OLED devices were also evaluated as a function of applied voltage at a fixed frequency of 1 kHz (Fig. 6e). They increased with applied voltage reaching maximum values of 1.4, 7.0, 14.3 and 4.9 cd/A for blue, cyan-blue, green and red devices, respectively.

As demonstrated for TPEL devices, the effect of AC frequency on the performance of devices is also significant. Therefore, we investigated the dependencies of frequency on luminance and current efficiency of TP-OLEDs (at a fixed voltage of 53 V) (Fig. 6g,h). For all TP-OLEDs, the luminance increased with a frequency, with saturation at high frequencies and approaching the maximum values at the frequencies above ca 2 kHz. No decrease in luminance was observed even at high frequencies of 10 kHz (in contrast to TPEL devices, Supplementary Fig. 6). The current efficiencies showed peak values around 500 – 2000 Hz, with dropping the efficiency at higher frequencies. (Fig. 6h).

Similar measurements were performed for green devices with a thickness of electron-transporting layer (ETL) of 70 nm. An increase of the ETL thickness showed somewhat better device performance (Supplementary Figs. 19, 21).

5. Supplementary Figures 1 – 21.

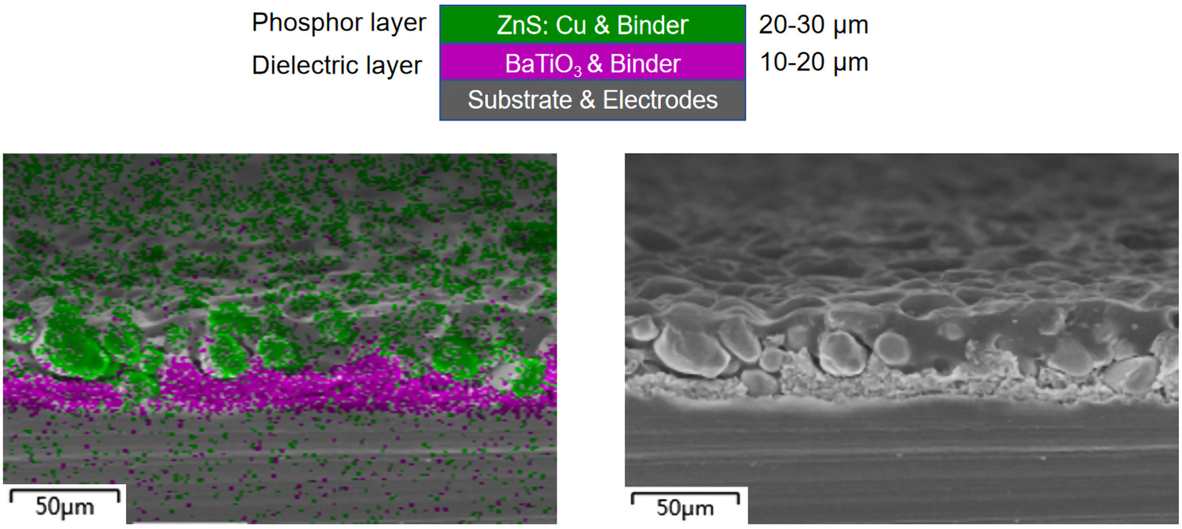

Supplementary Figure 1 | Cross-sectional scanning electron microscopy (SEM) image (right) and corresponding energy dispersive X-ray spectroscopy (EDS) map (left) of a TPEL device, with its schematic representation on the top. The colors on the EDS image correspond to that on the top scheme. The grey color in a phosphor layer is a binder. PET-ITO was used as substrate / electrodes. Commercial phosphor (GG65, Leuchtstoffwerk Breitungen GmbH) was used in in the light-emitting layer.

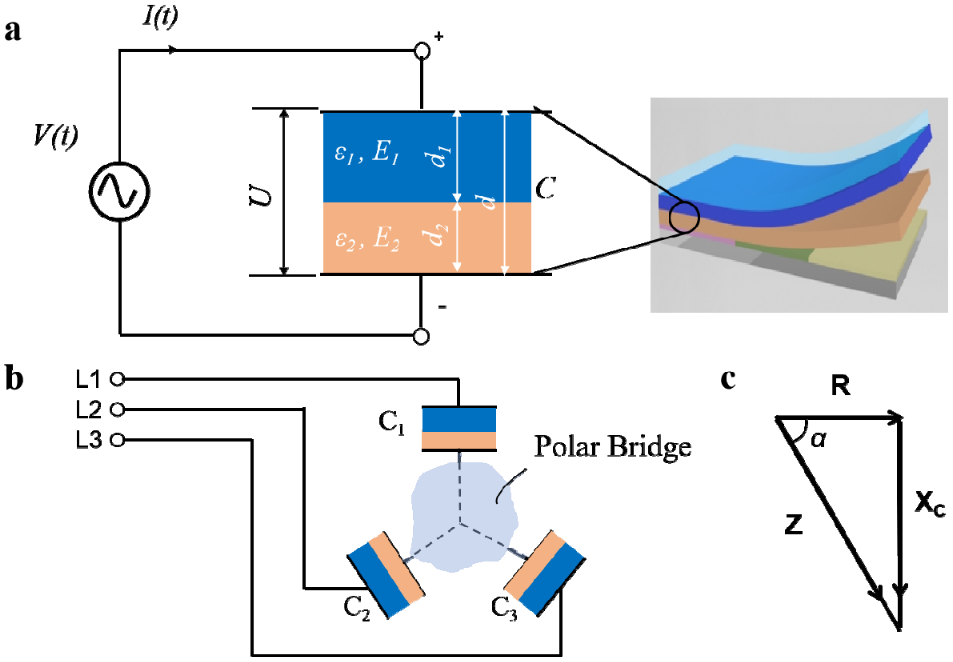

Supplementary Figure 2 | Schematic illustration of TPEL devices. a, Schematic illustration of two-layer dielectric model. **b,** Schematic illustration of typical three-phase three-wire equivalent circuit diagram for the connection of TPEL devices. It should be noted that only for pixel formed TPEL devices we need an extra electrode connected to the PEN (protective neutral) line and unless stated otherwise, we used three-phase three-wire system to drive TPEL devices. **c,** Schematic diagram of impedance triangle. Here, **R** is the impedance introduced by resistive component, $\mathbf{X_c}$ is the impedance introduced by capacitive component, **Z** is the total impedance and α indicates the angle of total impedance (that is the angle between the current and voltage in the circuit).

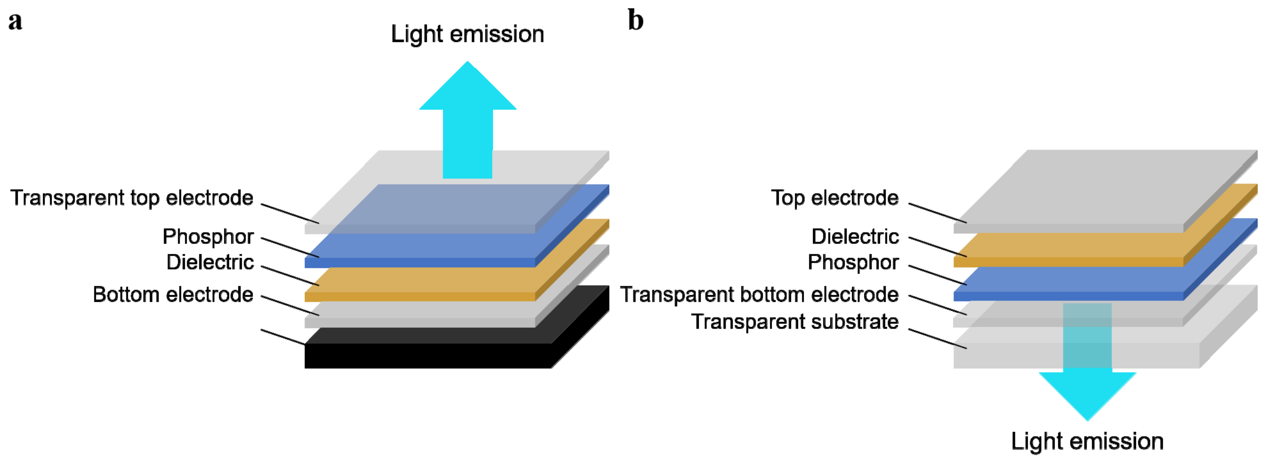

Supplementary Figure 3 | Illustration of two kinds of conventional AC driven electroluminance devices. **a,** top-emission structure (TES). **b,** bottom-emission structure (BES).

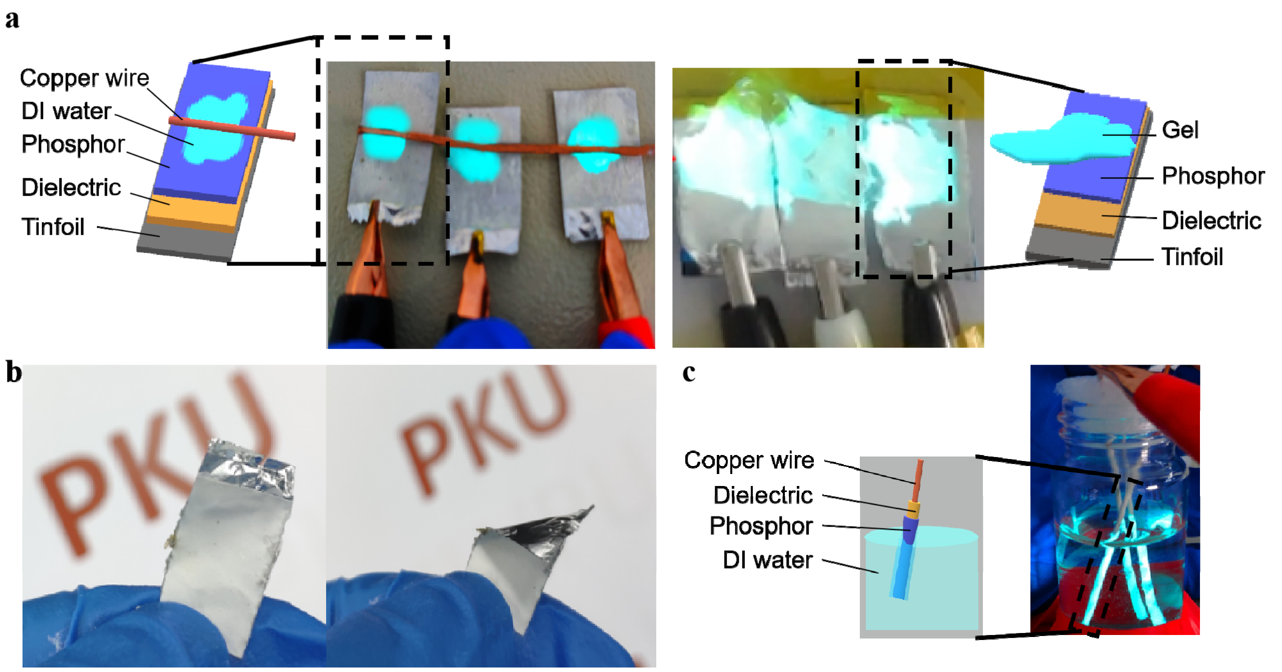

Supplementary Figure 4 | Simple TPEL devices with no substrate and ubiquitous electrodes. a, Photographs and inserted schematic structure of TPEL devices fabricated using tinfoil as an electrode (with no additional substrate on the bottom). DI water and copper wire (left) or hydrogel (right) were used as a polar electrode bridge between the lines. A blade coating method was used in this case and we did not deliberately control the thickness of each layer. **b,** Flexibility of the tinfoil based TPEL unit in (a). **c,** Photograph and inserted schematic cartoon of fibrous TPEL devices fabricated using copper wire as an electrode. DI water was used as a polar bridge. We used a dip coating method in this case without deliberately controlling the thickness of each layer.

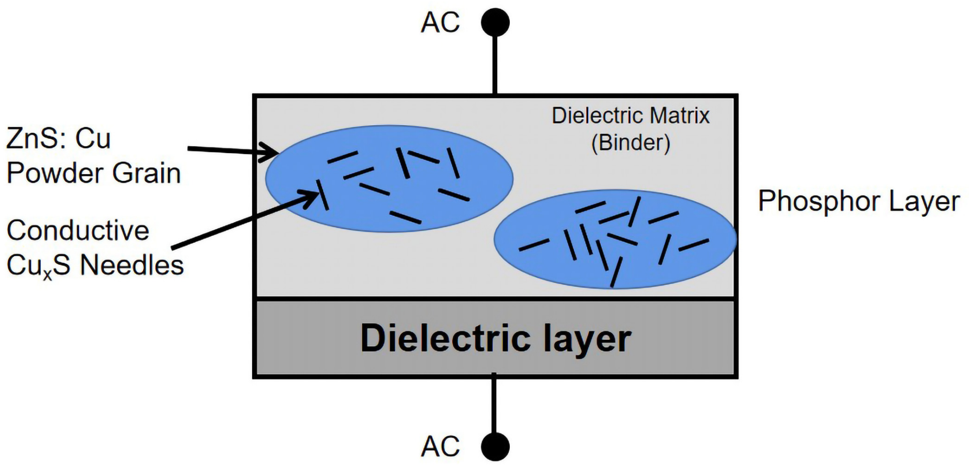

Supplementary Figure 5 | ZnS:Cu phosphor particle according to the Fischer model. Cu_xS needles are contained in the ZnS:Cu grains.

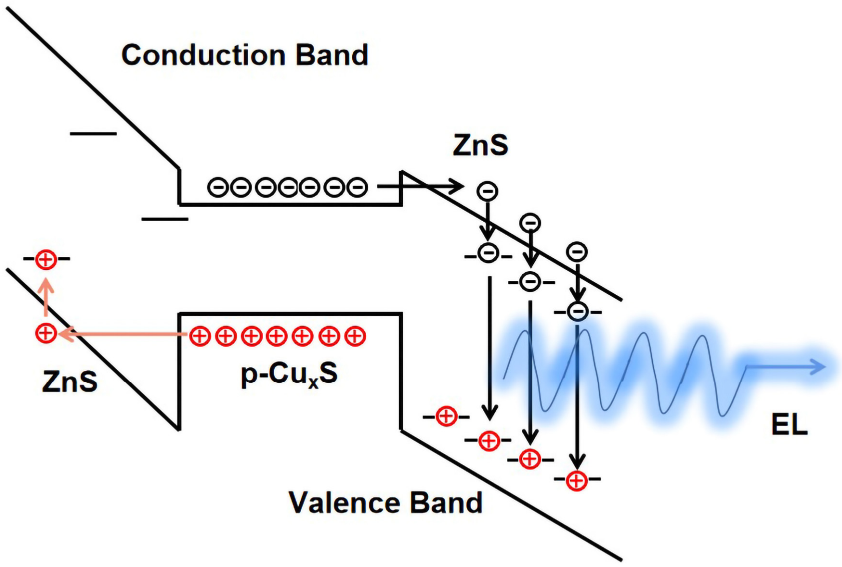

Supplementary Figure 6 | Schematic energy level diagram according to Fischer model. Energy level of phosphor layer for one of three capacitors E/Ph/PEB in a TPEL device was shown in this diagram.

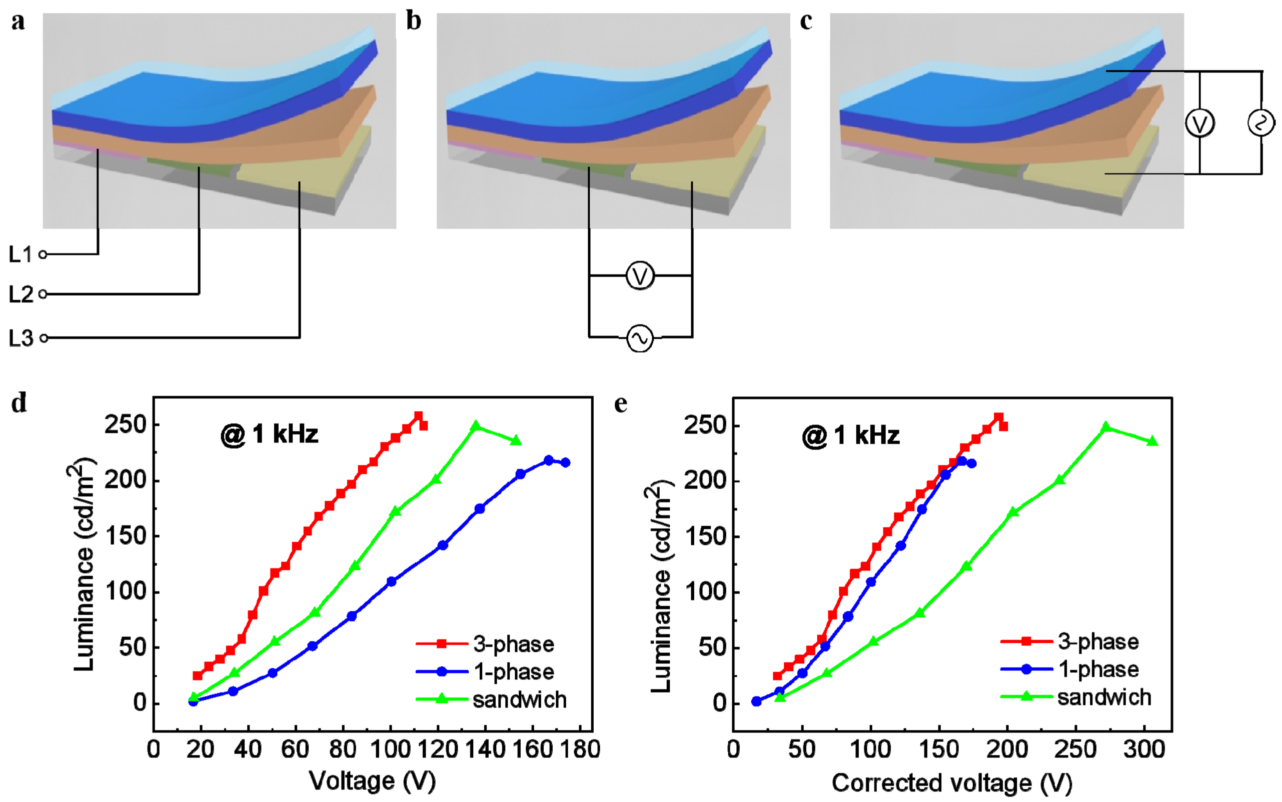

Supplementary Figure 7 | Luminance performance comparison of TPEL device driven by a three-phase, single-phase and sandwich electric system. Schematic diagram of a three-phase **(a)**, single-phase **(b)** and sandwich **(c)** electric system driven devices. **d,** Luminance performance comparison between the three driving systems using the same device, in which X axis represents the phase voltage for a three-phase system and voltmeter readings in (**b**) and (**c**) for single-phase and sandwich systems, respectively. **e,** Luminance performance comparison of the three driving systems versus the line voltage. For the three-phase electric system, the actual voltage between the two arbitrary electrodes is the line voltage. So, the X axis of the three-phase driving system was modified into the line voltage (multiplying the phase voltage by √3). For a single-phase driving system, the actual voltage between two electrodes is a phase voltage, so it was kept unchanged. For a sandwich driving system, we used a conductive tape connected to the polar bridge layer to build up the top electrode. The theoretical value of the voltage driven by a sandwich system should be about 1/2 of the voltage driven by a single-phase system under the same conditions, so the X axis of the sandwich driving system was modified by multiplying the phase voltage by 2 for proper comparison. Commercial bath gel was used to form the polar bridge in this part.
Source data are provided as a Source Data file.

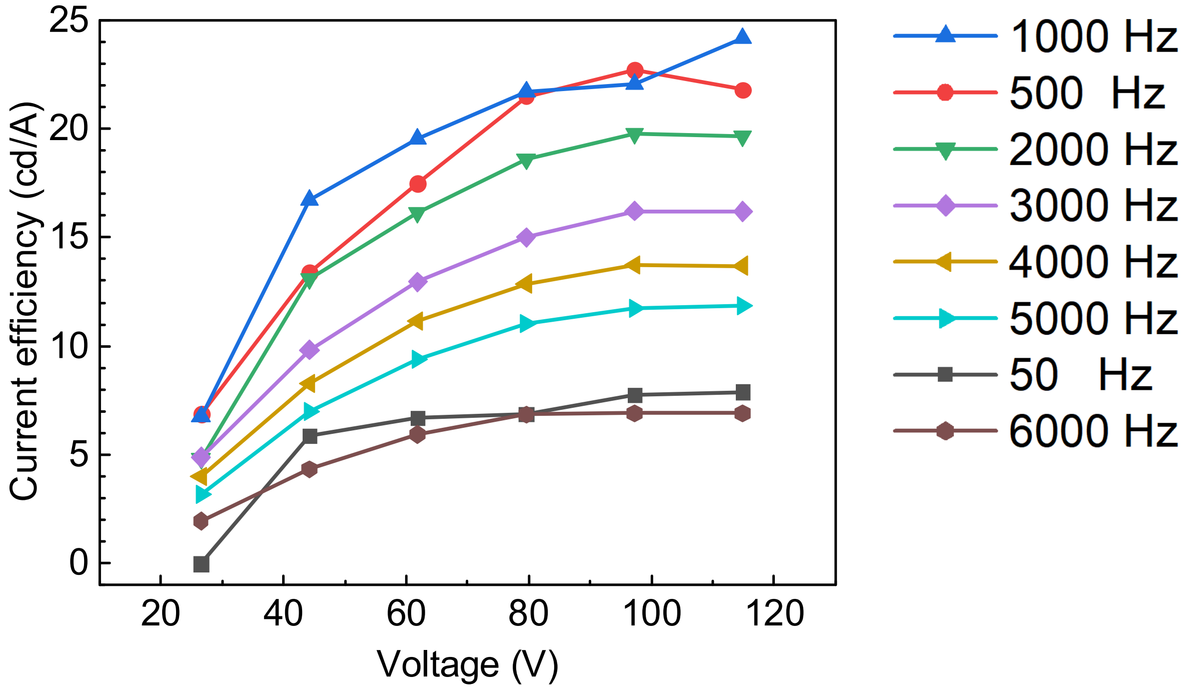

Supplementary Figure 8 | The relationships between the current efficiency and applied voltage at different frequencies for standard TPEL device using DI water as polar bridge. Glass-ITO was used as substrate / electrodes, with a light emitting area of 3 × 2 cm. Commercial phosphor (GG65, Leuchtstoffwerk Breitungen GmbH) was used in the light-emitting layer. The current efficiency is increased with an increase of the frequency to *ca.* 500 – 1000 Hz and then decreased.

Source data are provided as a Source Data file.

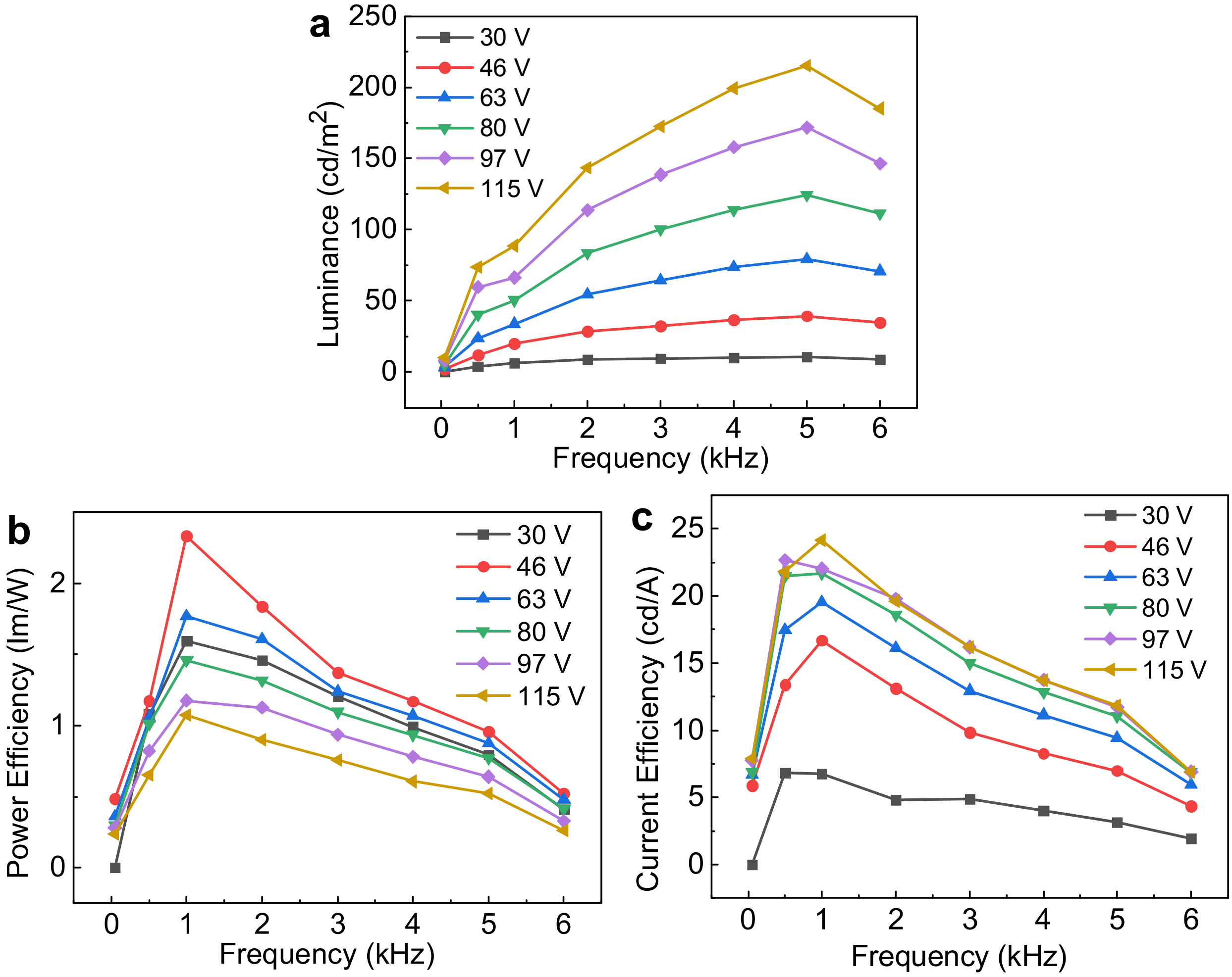

Supplementary Figure 9 | Effects of frequency on the performance of standard TPEL device using DI water as a polar bridge. Dependence of luminance **(a)**, power efficiency **(b)** and current efficiency **(c)** on the AC frequency at different voltages. DI water was used as a polar bridge. Glass-ITO was used as substrate / electrodes, with a light emitting area of 3 × 2 cm. Commercial phosphor (GG65, Leuchtstoffwerk Breitungen GmbH) was used in the light-emitting layer.

Source data are provided as a Source Data file.

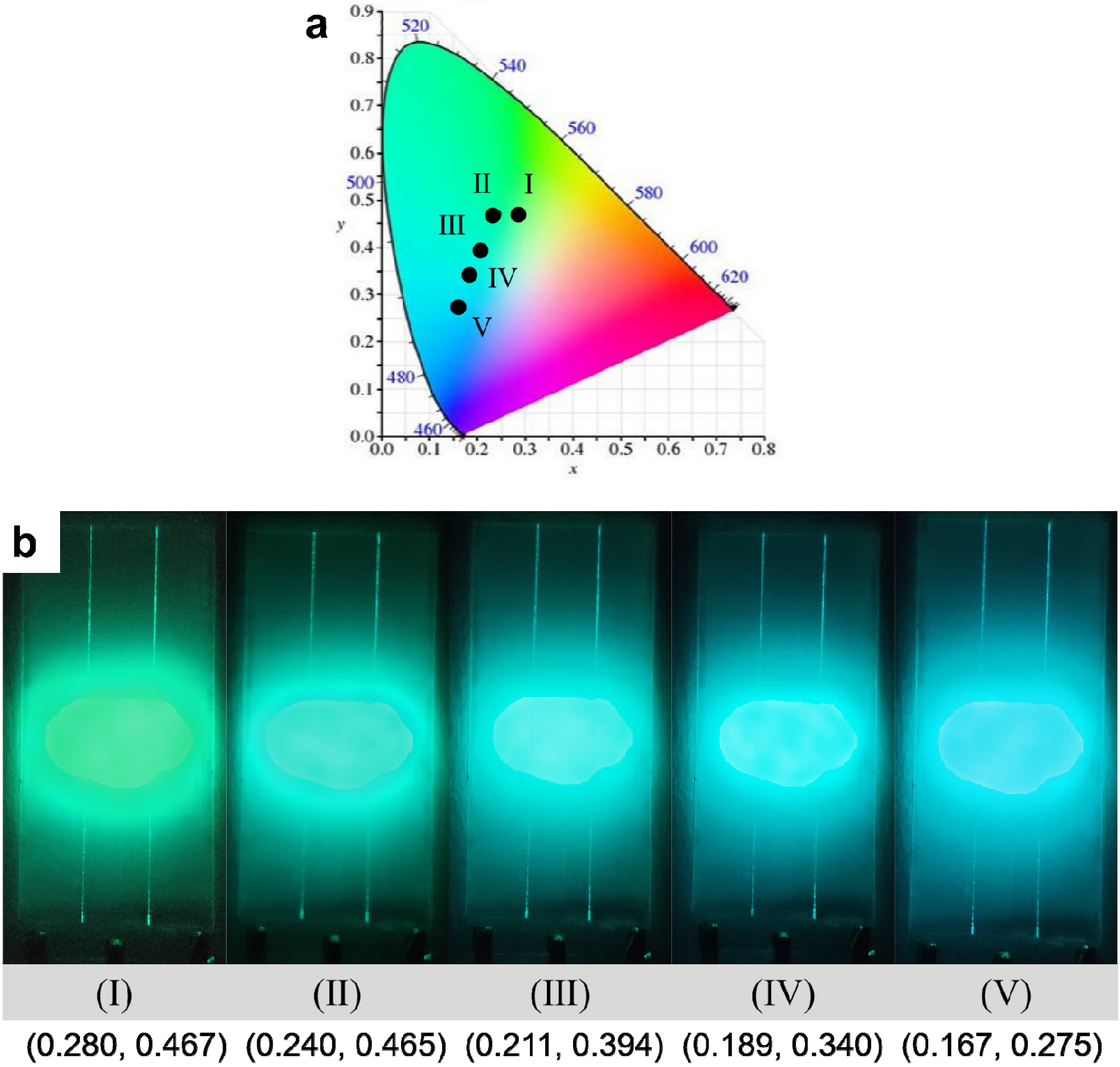

Supplementary Figure 10 | The effect of AC frequency on the EL emission of TPEL devices with GC65 phosphor. a, CIE (x,y) chromaticity diagram of the EL emission of TPEL devices driven by 80 V AC voltage at 50 Hz (I), 100Hz (II) 300 Hz (III), 500 Hz (IV) and 1000 Hz (V), respectively. I – V were all fabricated using commercial phosphor (GG65, Leuchtstoffwerk Breitungen GmbH) as an emitting layer and DI water as a polar bridge. **b,** Photographs and CIE (x,y) coordinates of corresponding light emissions of I – V TPEL devices (2 × 4 cm, with shown light emission from the area of *ca.* 1 cm^2).

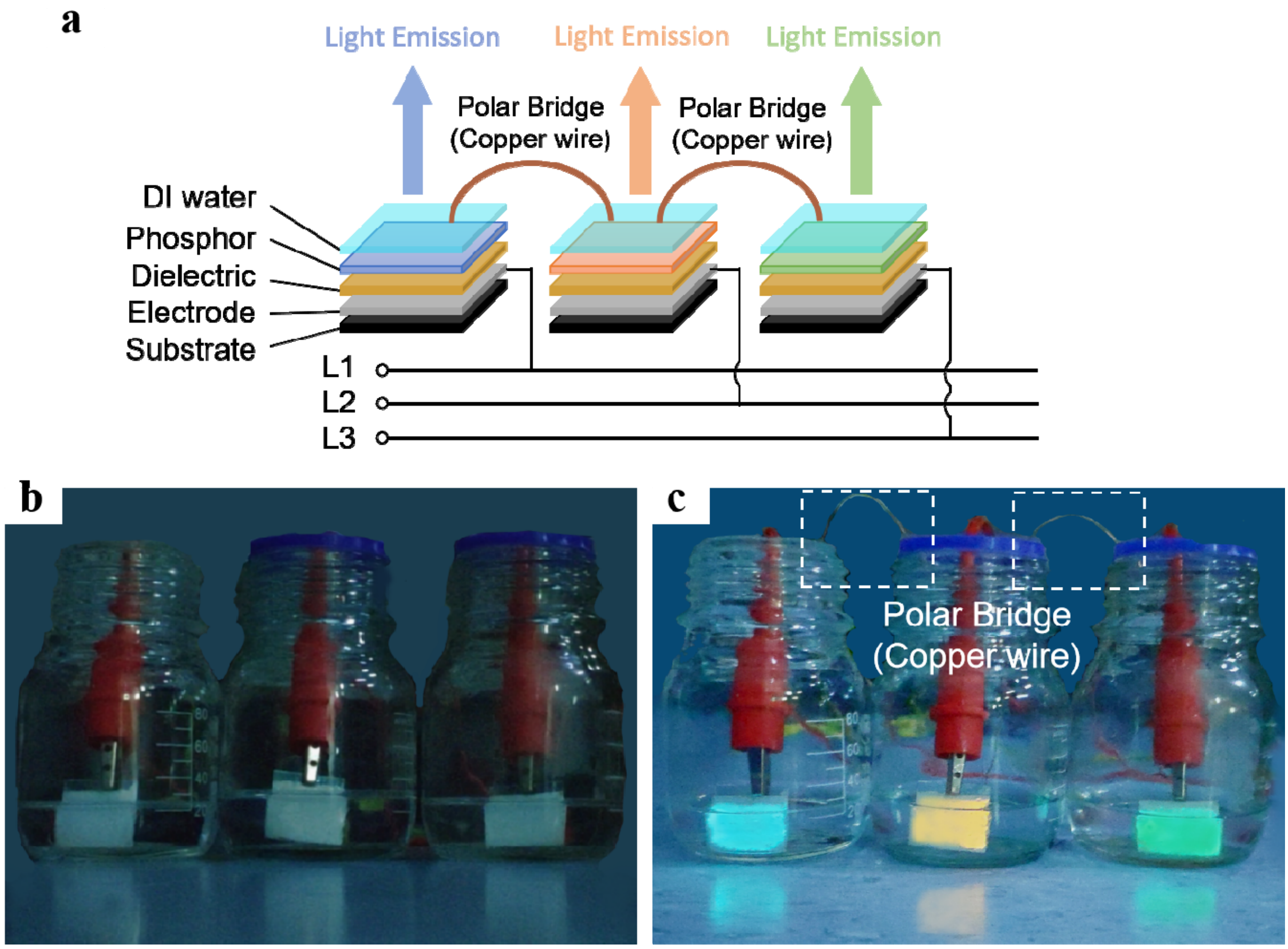

Supplementary Figure 11 | Polar bridge experiments using DI water and conductive copper wire as a polar bridge. a, Schematic diagram of TPEL device separated into three parts, each driven by one single-phase AC supply of the three-phase electric power and connected together by a polar bridge. Here we used DI water as polar bridge in each part, which are connected by copper wires. **b,c,** The photographs of three separate units placed into the beakers with DI water, each of them is connected to one single-phase AC supply of the three-phases electric power: **(b)** without copper electrode bridges between the units (<u>no light emission</u>), **(c)** copper wires are used as bridges (<u>each unit emits the light from its phosphor layer</u>). The phosphors used as emissive layers on **(b,c)** are GG65, GG14 and GG45 (purchased from Leuchtstoffwerk Breitungen GmbH) from the left to the right. PET-ITO was used as substrate / electrode.

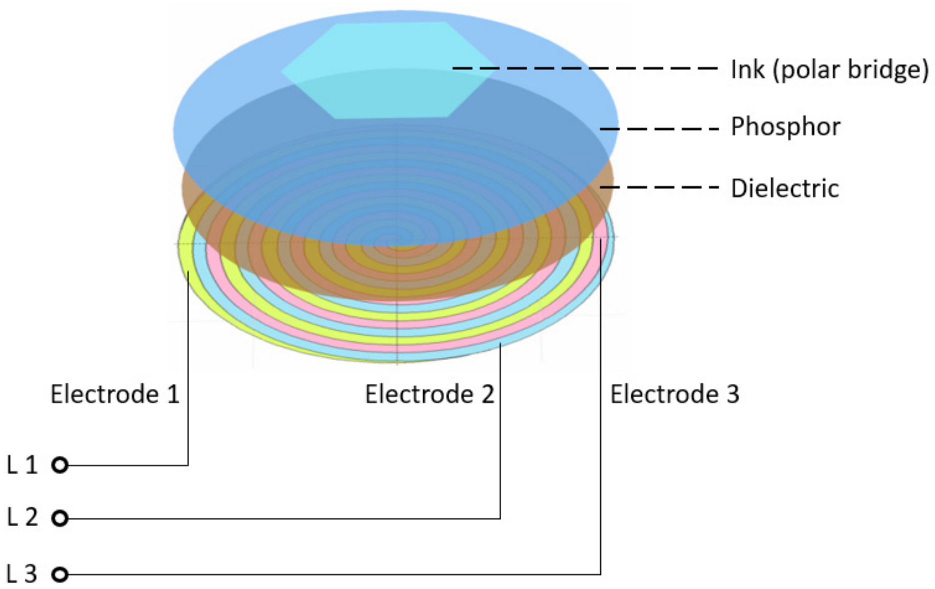

Supplementary Figure 12 | Schematic diagram of TPEL panel (lollipop-type electrode is shown as an example). The conductive ITO film on PET substrate was divided into three uniform parts and three separated electrodes are shown in the schematic diagram as green, red and blue. It should be noted that we used ITO as electrodes, so all three electrodes are transparent. Three different colors are used in the schematic diagram just to distinguish the different electrodes visually. A three-phase three-wire system was used to drive the TPEL panel. Three electrodes were connected to three different live wires, respectively. DI water, hydrogel, water-based fluorescent pen or graphite pencil can be used to provide ink (acting as a polar electrode bridge) to create arbitrary light emitting pattern. Thus, only ink-covered areas will emit the light.

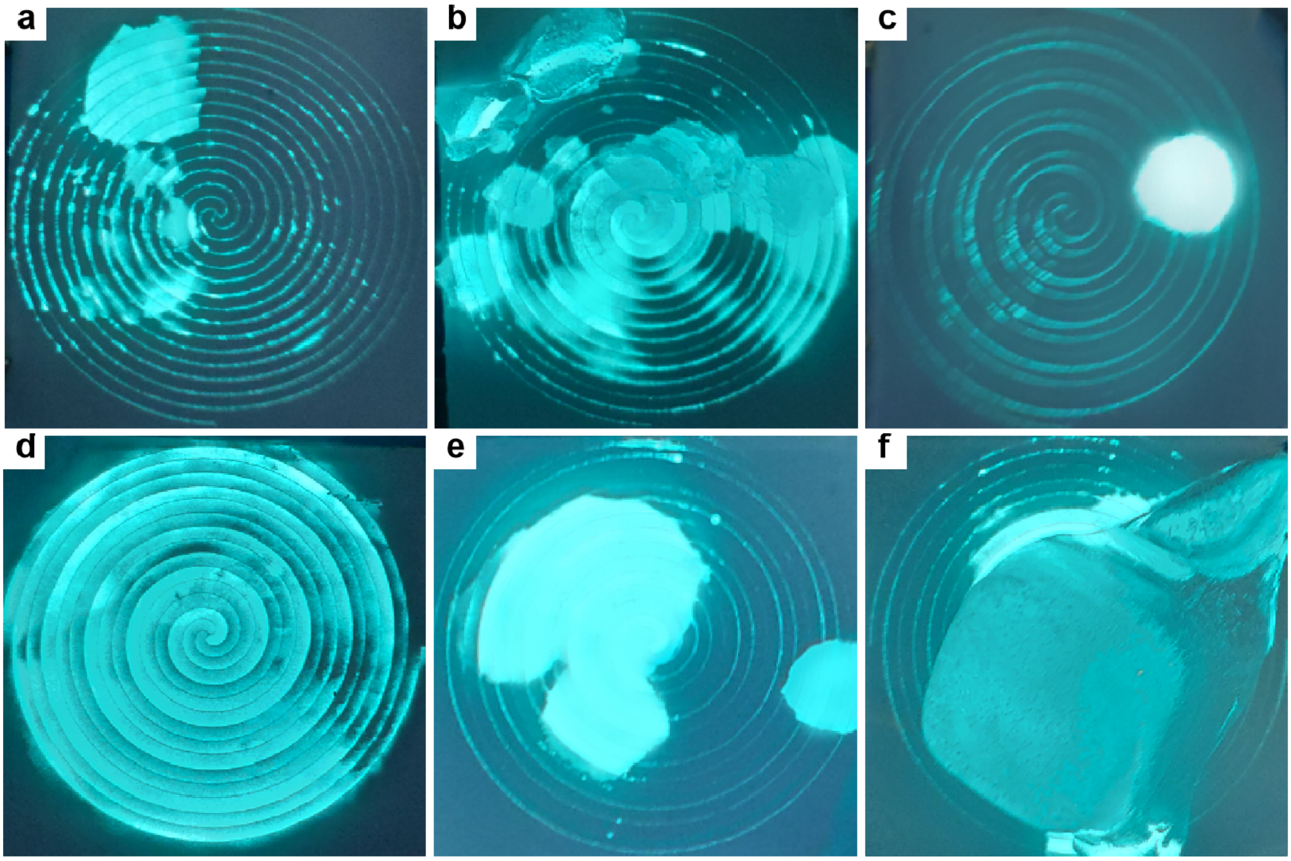

Supplementary Figure 13 | Photographs of multi-functional TPEL panels using lollipop-type electrodes (schematic diagram of which is shown in Supplementary Fig. 12), applied to the simulated environment of **(a)** snow, **(b)** frozen rain, **(c)** dew, **(d)** extremely wet environment (heavy fog, relative humidity 100%), **(e)** rain and **(f)** ice accretion. TPEL panels respond strongly to all these extreme environments starting to emit the light from the phosphor and raise an optical alarm remotely.

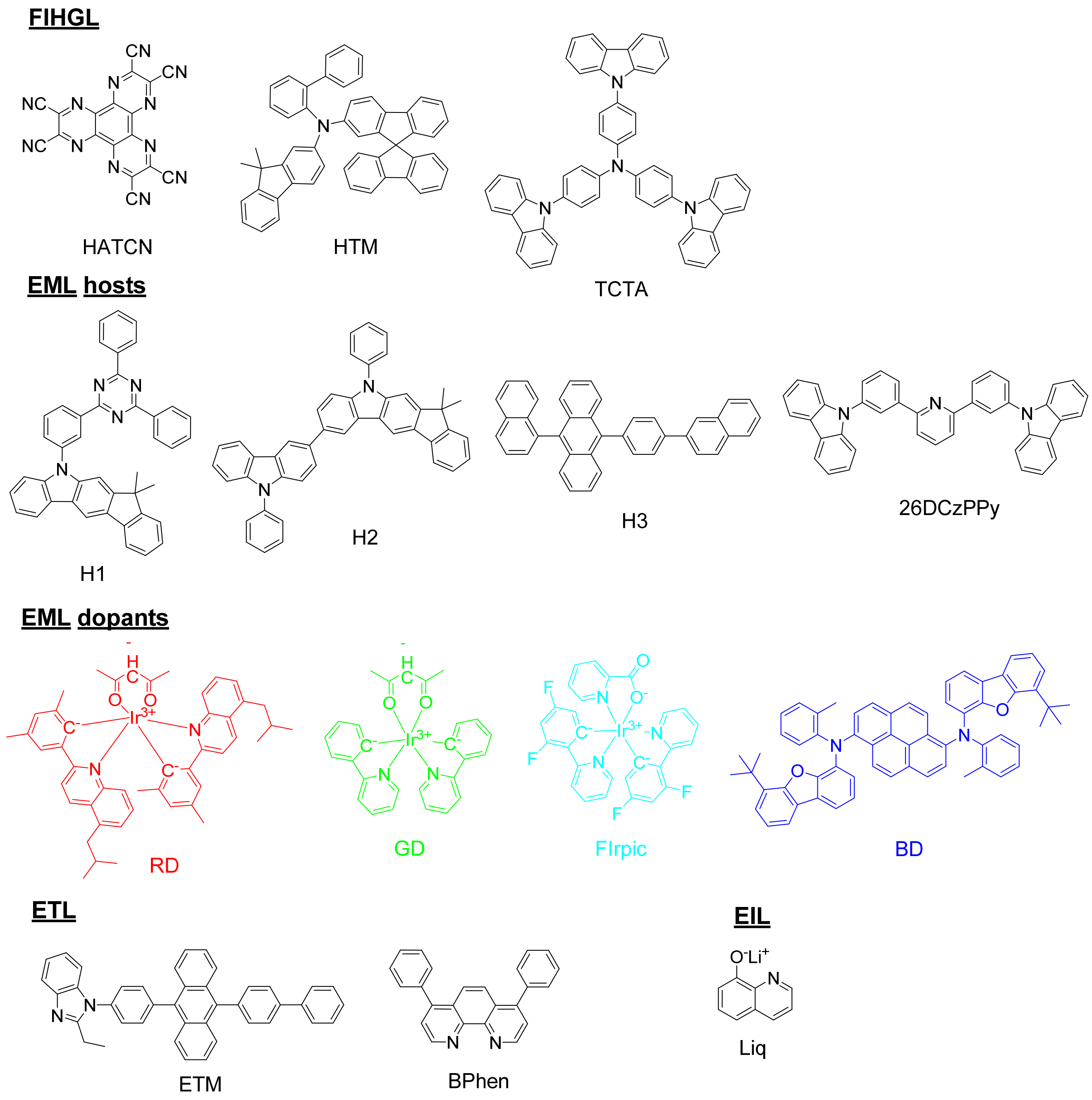

Supplementary Figure 14 | Chemical structures and acronyms for compounds used in fabrication of TP-OLEDs. All of the above compounds are deposited by a vacuum thermal evaporation method to fabricate TP-OLEDs.

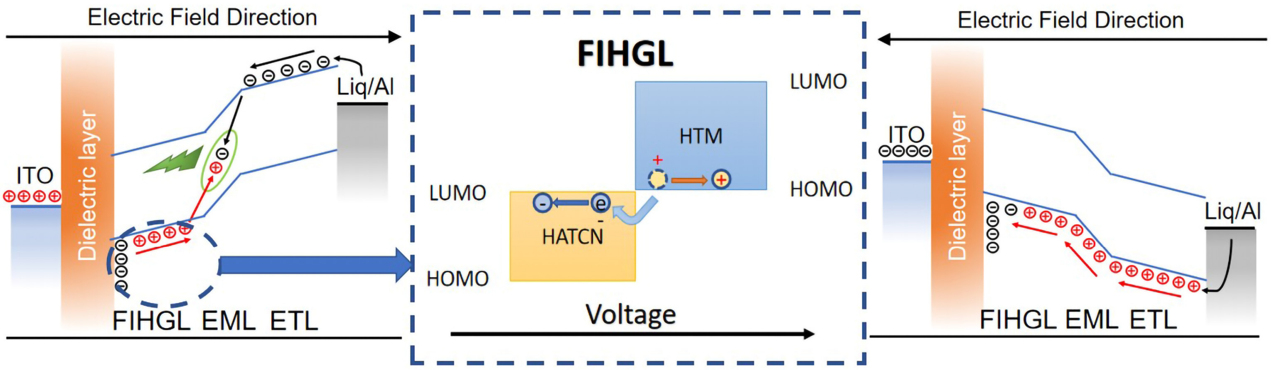

Supplementary Figure 15 | Schematic representation of both half-cycles (left and right graphs) of an AC excitation in one of the three capacitors E(–)//EML//EB(+) of TP-OLED (the structure of which is shown in Fig. 6a in the main text). The middle graph schematically shows the energy level structure of FIGHL consisting electron acceptor (HATCN) and electron donor (HTM) layers at the interface of which field induced charge generation occurs.

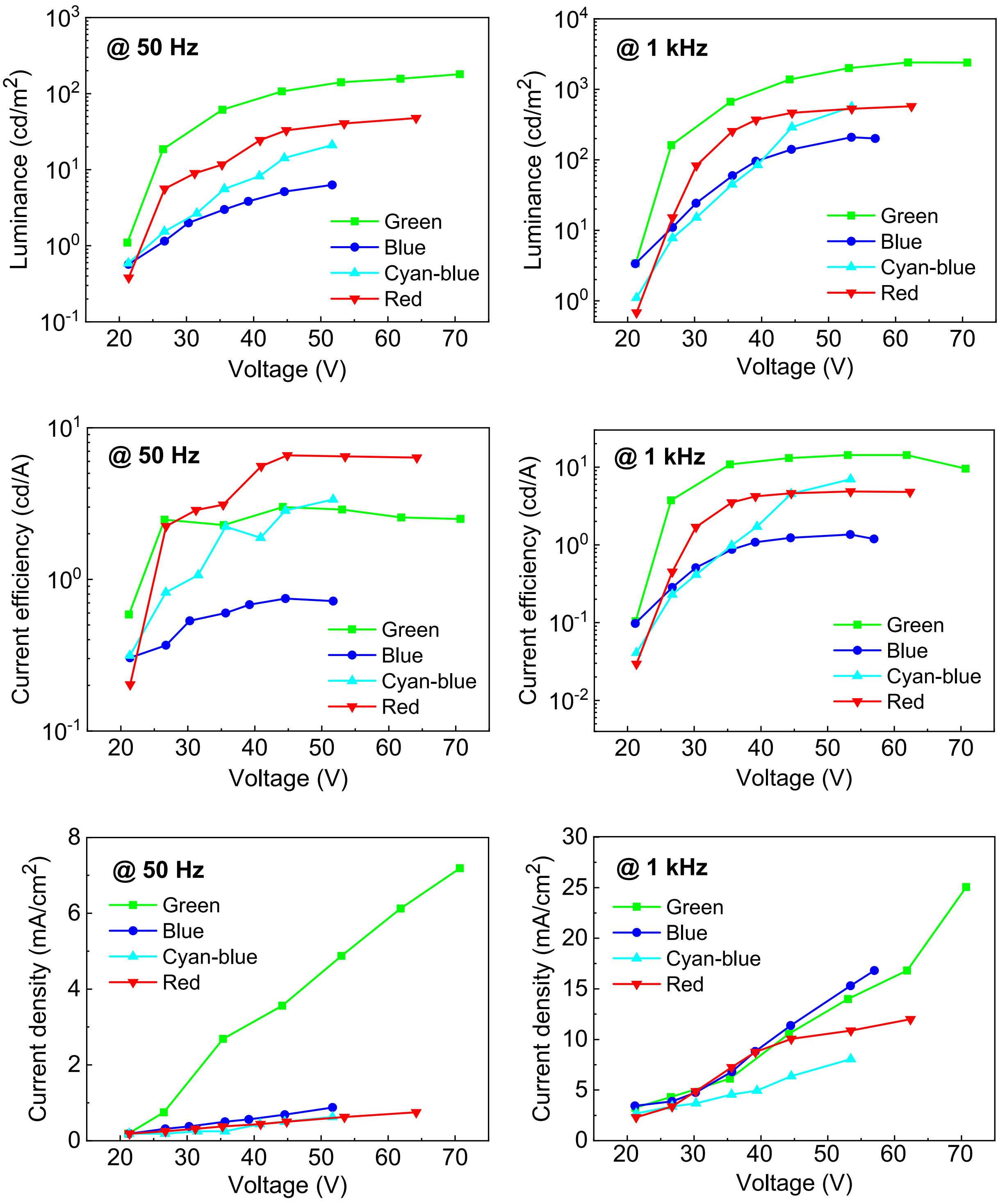

Supplementary Figure 16 | Luminance, current efficiency and current density of standard TP-OLEDs as functions of voltage at the fixed frequencies of 50 Hz (left) and 1 kHz (right).

Green: HATCN (10 nm) / HTM (30 nm) / 48.8% H1 : 48.8% H2 : 2.4% GD (50 nm) / ETM (30 nm) / Liq (2.5 nm) / Al (100 nm)

Blue: HATCN (10 nm) / HTM (30 nm) / 90.9% H3 : 9.1% BD (50 nm) / ETM (30 nm) / Liq (2.5 nm) / Al (100 nm)

Cyan-blue: HATCN (10 nm) / TCTA (40 nm) / 90.9% 26DCzPPy : 9.1% FIrpic (50 nm) / BPhen (40 nm) / Liq (2.5 nm) / Al (100 nm)

Red: HATCN (10 nm) / HTM (30 nm) / 65% H1 : 32.5% H2 : 2.5% RD (40 nm) / ETM (30 nm) / Liq (2.5 nm) / Al (100 nm)

Source data are provided as a Source Data file.

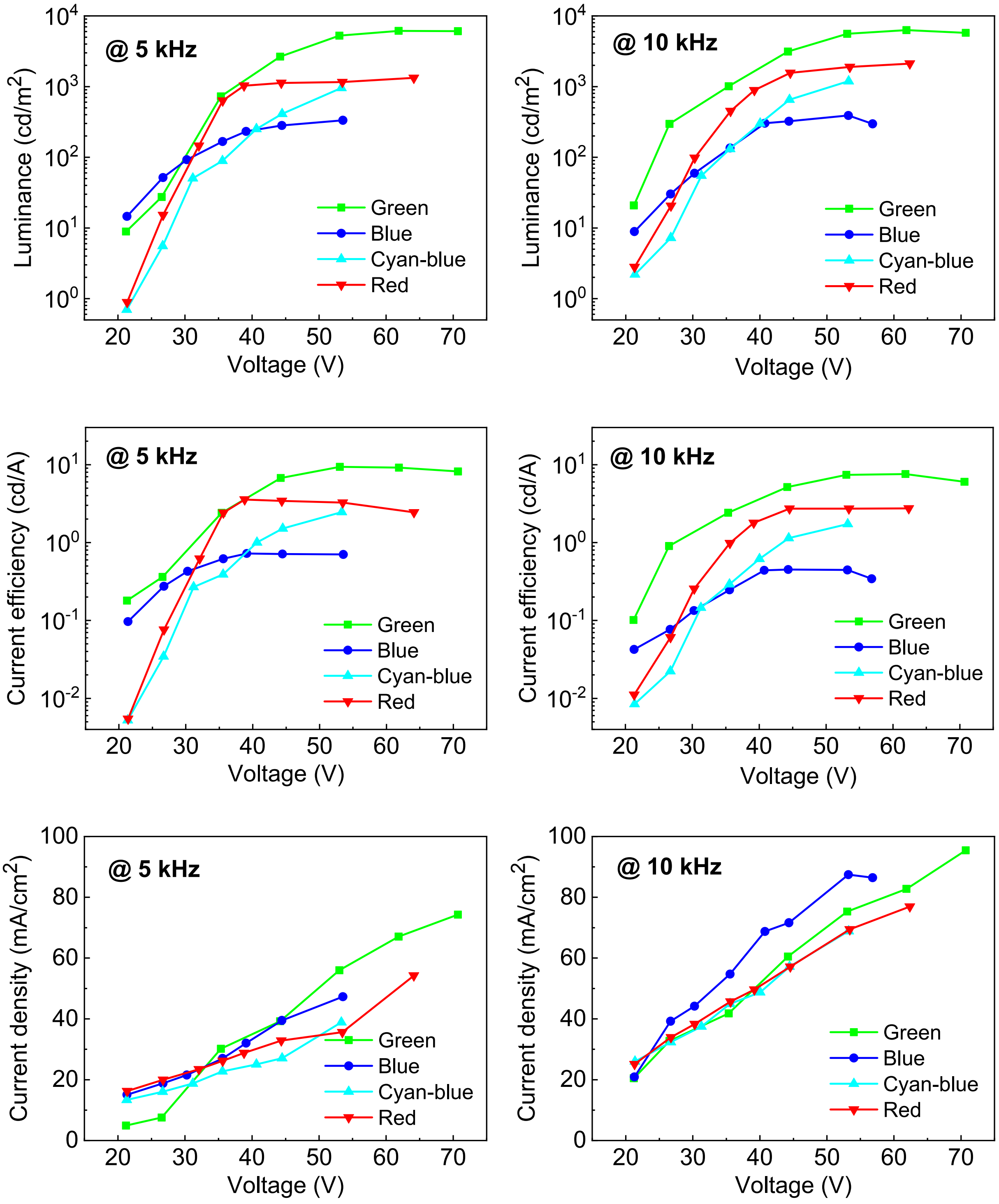

Supplementary Figure 17 | Luminance, current efficiency and current density of standard TP-OLEDs as functions of voltage at the fixed frequencies of 5 kHz (left) and 10 kHz (right).

Green: HATCN (10 nm) / HTM (30 nm) / 48.8% H1 : 48.8% H2 : 2.4% GD (50 nm) / ETM (30 nm) / Liq (2.5 nm) / Al (100 nm)

Blue: HATCN (10 nm) / HTM (30 nm) / 90.9% H3 : 9.1% BD (50 nm) / ETM (30 nm) / Liq (2.5 nm) / Al (100 nm)

Cyan-blue: HATCN (10 nm) / TCTA (40 nm) / 90.9% 26DCzPPy : 9.1% FIrpic (50 nm) / BPhen (40 nm) / Liq (2.5 nm) / Al (100 nm)

Red: HATCN (10 nm) / HTM (30 nm) / 65% H1 : 32.5% H2 : 2.5% RD (40 nm) / ETM (30 nm) / Liq (2.5 nm) / Al (100 nm)

Source data are provided as a Source Data file.

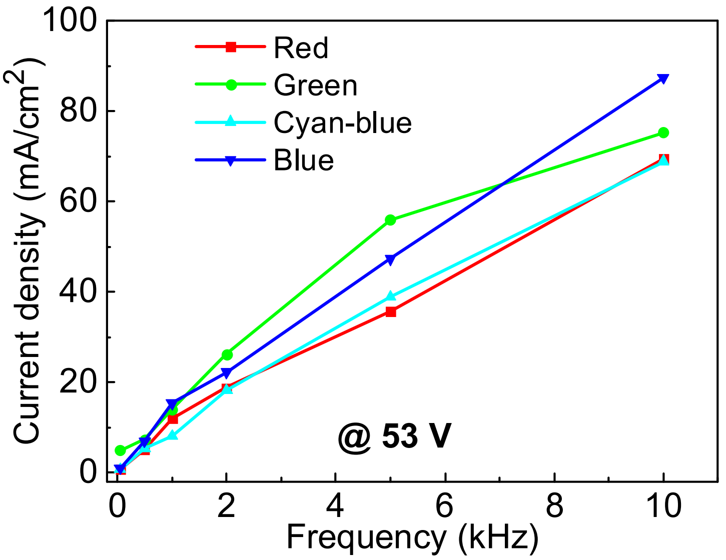

Supplementary Figure 18 | Current density of standard TP-OLEDs as functions of AC frequency at the fixed voltage of 53 V.

Green: HATCN (10 nm) / HTM (30 nm) / 48.8% H1 : 48.8% H2 : 2.4% GD (50 nm) / ETM (30 nm) / Liq (2.5 nm) / Al (100 nm)

Blue: HATCN (10 nm) / HTM (30 nm) / 90.9% H3 : 9.1% BD (50 nm) / ETM (30 nm) / Liq (2.5 nm) / Al (100 nm)

Cyan-blue: HATCN (10 nm) / TCTA (40 nm) / 90.9% 26DCzPPy : 9.1% FIrpic (50 nm) / BPhen (40 nm) / Liq (2.5 nm) / Al (100 nm)

Red: HATCN (10 nm) / HTM (30 nm) / 65% H1 : 32.5% H2 : 2.5% RD (40 nm) / ETM (30 nm) / Liq (2.5 nm) / Al (100 nm)

Source data are provided as a Source Data file.

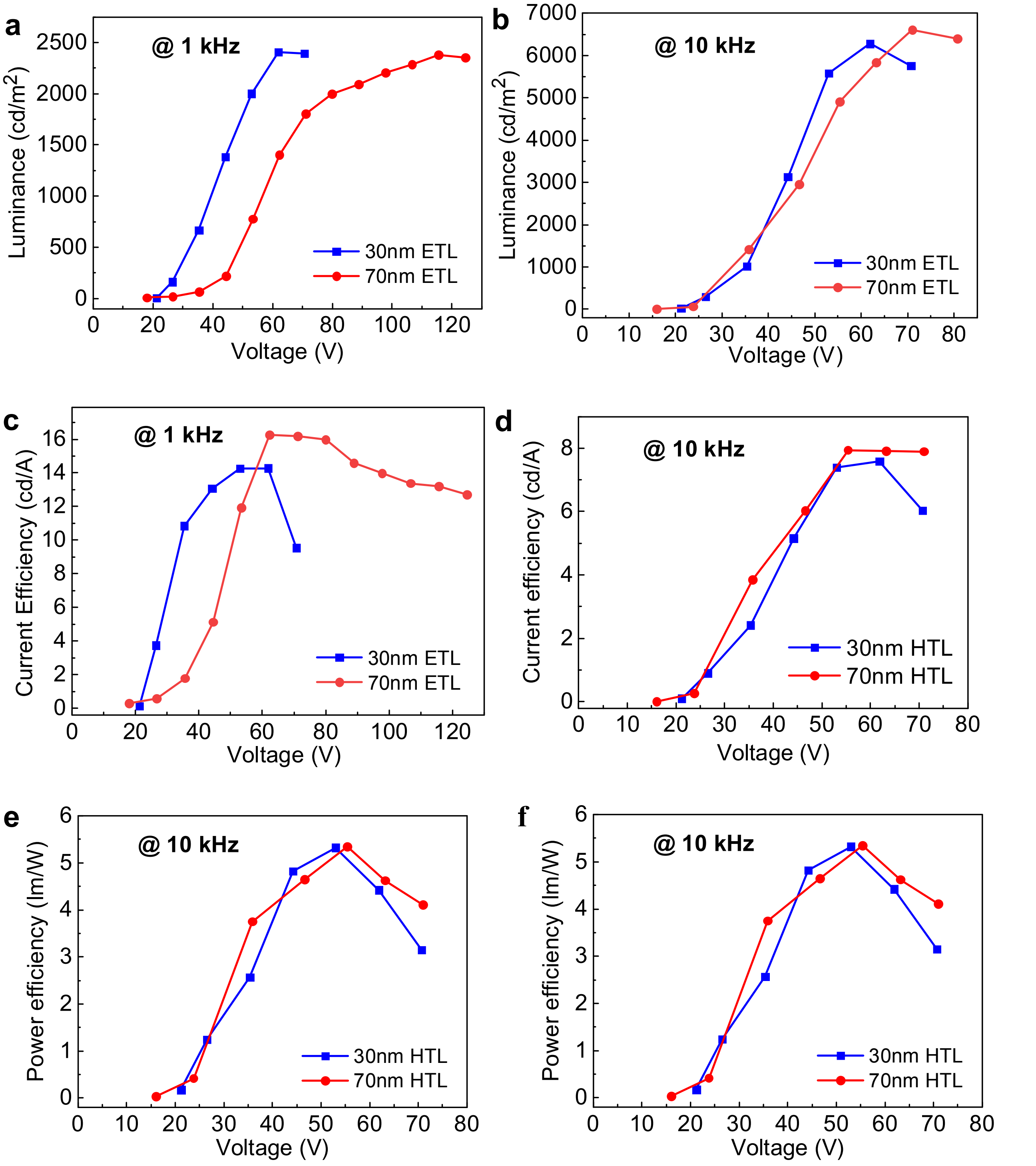

Supplementary Figure 19 | Luminance (a,b), current efficiency (c,d) and power efficiency (e,f) performance characteristics of green TP-OLED with different thickness of ETL layers at fixed frequencies of 1 kHz (a,c,e) and 10 kHz (b,d,f). Device structure:

HATCN(10 nm) / HTM(30 nm) / 48.8%H1 : 48.8%H2 : 2.4%GD (50 nm) / ETM(30 or 70 nm) / Liq(2.5 nm) / Al(100 nm)

30 nm ETL device:

L_{max} = 6277 cd/m^2 (at 71 V / 10 kHz; C); CE_{max} = 14.3 cd/A (at 62 V / 1 kHz; B); PE_{max} = 13.9 lm/W (at 44 V / 1 kHz; E);

70 nm ETL device:

L_{max} = 6601 cd/m^2 (at 71 V / 10 kHz; C); CE_{max} = 16.2 cd/A (at 62 V / 1 kHz; B); PE_{max} = 17.0 lm/W (at 62 V / 1 kHz; E).

Source data are provided as a Source Data file.

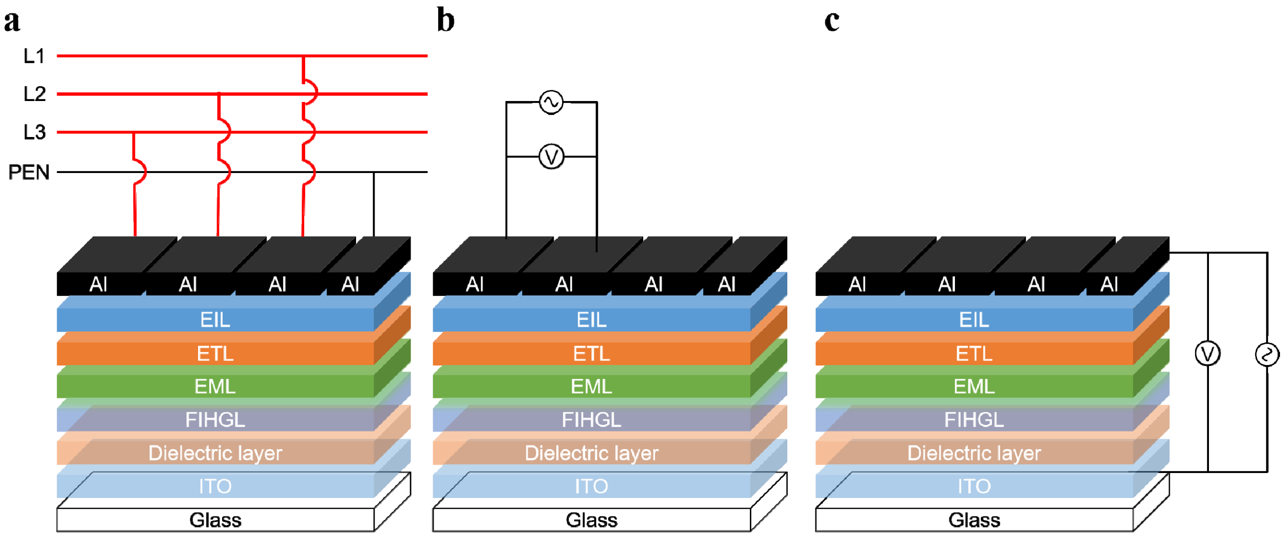

Supplementary Figure 20 | Schematic diagram of a single-insulating AC OLED device driven by a three-phase (a), single-phase (b) and sandwich (c) electric system.

Comparative performance of the device is shown in Figure S21.

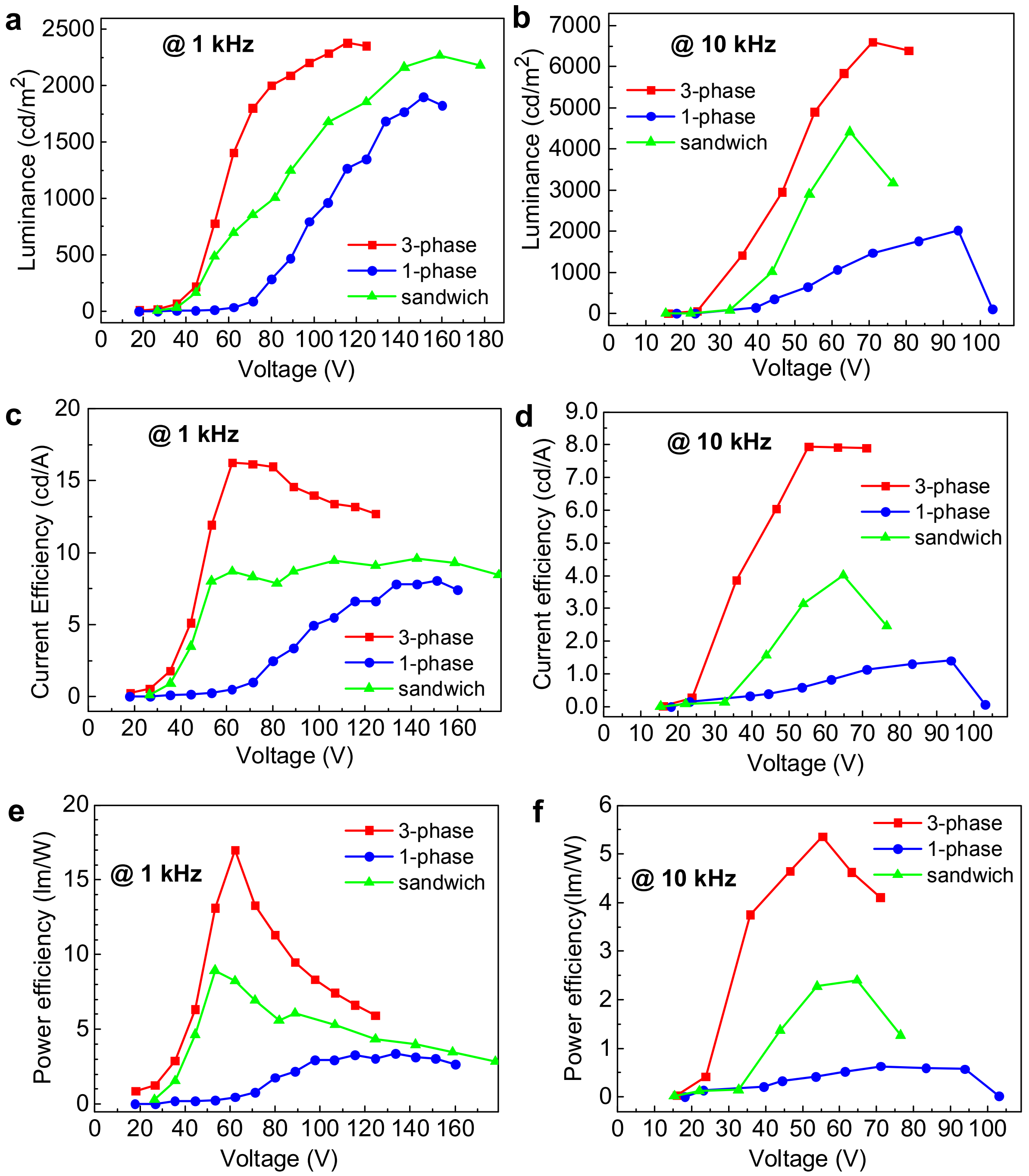

Supplementary Figure 21 | Performance comparison of green single-insulating AC OLED device driven by a three-phase, single-phase and sandwich electric system. (Schematic diagrams of the devices are shown in Figure S20). Luminance **(a,b)**, current efficiency **(c,d)** and power efficiency **(e,f)** performance comparison between the three driving systems using the same device at fixed frequencies of 1 kHz (**a,c,e**) and 10 kHz (**b,d,f**). X axes represent the phase voltage for three-phase system and voltmeter readings for single-phase and sandwich systems (see Figure 20), respectively. Device structure:

HATCN(10 nm) / HTM(30 nm) / 48.8%H1 : 48.8%H2 : 2.4%GD (50 nm) / ETM(70 nm) / Liq(2.5 nm) / Al(100 nm)

3-phase: L_{max} = 6601 cd/m² (at 71 V / 10 kHz; B); CE_{max} = 16.2 cd/A (at 62 V / 1 kHz; C); PE_{max} = 17.0 lm/W (at 62 V / 1 kHz; E);

1-phase: L_{max} = 4416 cd/m² (at 65 V / 10 kHz; B); CE_{max} = 9.6 cd/A (at 142 V / 1 kHz; C); PE_{max} = 3.4 lm/W (at 133 V / 1 kHz; E);

sandwich: L_{max} = 2019 cd/m² (at 94 V / 10 kHz; F); CE_{max} = 8.1 cd/A (at 151 V / 1 kHz; E); PE_{max} = 8.9 lm/W (at 53V / 1 kHz; E).

Source data are provided as a Source Data file.
