# Supplementary material for: Three-phase electric power driven electoluminescent devices
Source: Nat Commun. 2021 Jan 4;12:54. doi: 10.1038/s41467-020-20265-2 (PMC7782587; doi:10.1038/s41467-020-20265-2)
Supplement: Supplementary file 3 — Description of Additional Supplementary Files [file 41467_2020_20265_MOESM3_ESM.pdf]

Description of Additional Supplementary Files

File Name: Supplementary Movie 1
Description: TPEL device using tin foils as electrodes and hydrogel as a polar bridge.

File Name: Supplementary Movie 2
Description: TPEL device with independently controlled pixels to realize their different states.

File Name: Supplementary Movie 3
Description: Separated lighting EL units driven by three-phase electric power using hydrogel bridges with poor conductivity.

File Name: Supplementary Movie 4
Description: Separated EL units driven by three-phase electric power using copper wire accompanied with commercial hydrogel as a polar bridge.

File Name: Supplementary Movie 5
Description: Rewritable interactive display driven by three-phase electric power with writing-erasing capabilities.

File Name: Supplementary Movie 6
Description: TP-OLEDs with independently controlled pixels to realize their different states.
